# (*S*)-Methyl 3-(3,4-dimeth­oxy­phen­yl)-2-[2-(diphenyl­phosphan­yl)benzamido]­propano­ate

**DOI:** 10.1107/S1600536811047179

**Published:** 2011-11-12

**Authors:** Tricia Naicker, Thavendran Govender, Hendrick G. Kruger, Glenn E. M. Maguire

**Affiliations:** a School of Pharmacy and Pharmacology, University of KwaZulu-Natal, Durban 4000, South Africa; b School of Chemistry, University of KwaZulu-Natal, Durban 4000, South Africa

## Abstract

Mol­ecules of the title compound, C_31_H_30_NO_5_P, show a sttagered conformation about the C—C bond joining the dimeth­oxy­benzene group to the chiral centre, with the dimeth­oxy­benzene ring *gauche* to the amide group and *anti* to the ester group. In the crystal, weak inter­molecular N—H⋯O and C—H⋯O hydrogen bonds form layers parallel to (110).

## Related literature

For related structures, see: Clegg & Elsegood, (2003 ▶). For organocatalysts prepared from a related precursor, see: Naicker *et al.* (2010 ▶, 2011 ▶). For analogous precusors to several biologically active compounds, see: Zalán *et al.* (2006 ▶).
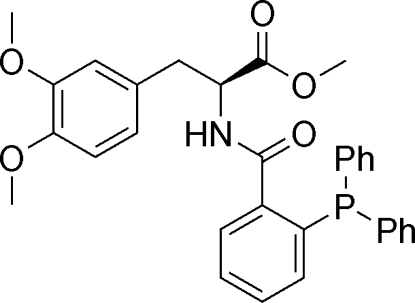


## Experimental

### 

#### Crystal data

C_31_H_30_NO_5_P
*M* *_r_* = 527.53
Monoclinic, 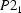

*a* = 10.2218 (3) Å
*b* = 8.4535 (2) Å
*c* = 15.7633 (4) Å
β = 100.300 (2)°
*V* = 1340.16 (6) Å^3^
*Z* = 2
Mo *K*α radiation
μ = 0.14 mm^−1^
*T* = 173 K
0.18 × 0.15 × 0.14 mm

#### Data collection

Nonius KappaCCD diffractometer
6654 measured reflections
6654 independent reflections
5550 reflections with *I* > 2σ(*I*)

#### Refinement

*R*[*F* ^2^ > 2σ(*F* ^2^)] = 0.034
*wR*(*F* ^2^) = 0.079
*S* = 1.04
6654 reflections
350 parameters
1 restraint
H atoms treated by a mixture of independent and constrained refinement
Δρ_max_ = 0.17 e Å^−3^
Δρ_min_ = −0.25 e Å^−3^
Absolute structure: Flack (1983 ▶), 3108 Friedel pairs
Flack parameter: −0.08 (6)

### 

Data collection: *COLLECT* (Nonius, 2000 ▶); cell refinement: *DENZO-SMN* (Otwinowski & Minor, 1997 ▶); data reduction: *DENZO-SMN*; program(s) used to solve structure: *SHELXS97* (Sheldrick, 2008 ▶); program(s) used to refine structure: *SHELXL97* (Sheldrick, 2008 ▶); molecular graphics: *OLEX2* (Dolomanov *et al.*, 2009 ▶); software used to prepare material for publication: *SHELXL97*.

## Supplementary Material

Crystal structure: contains datablock(s) I, global. DOI: 10.1107/S1600536811047179/lr2034sup1.cif

Structure factors: contains datablock(s) I. DOI: 10.1107/S1600536811047179/lr2034Isup2.hkl

Supplementary material file. DOI: 10.1107/S1600536811047179/lr2034Isup3.cml

Additional supplementary materials:  crystallographic information; 3D view; checkCIF report

## Figures and Tables

**Table 1:** Hydrogen-bond geometry (Å, °)

*D*—H⋯*A*	*D*—H	H⋯*A*	*D*⋯*A*	*D*—H⋯*A*
N1—H1*N*⋯O2^i^	0.816 (17)	2.345 (17)	3.1428 (17)	166 (15)
C10—H10*A*⋯O3^i^	0.98	2.56	3.371 (2)	140
C21—H21⋯O4^ii^	0.95	2.58	3.279 (2)	131

Symmetry codes: (i) 
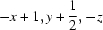
; (ii) 
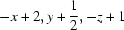
.

**Fig. 1.**
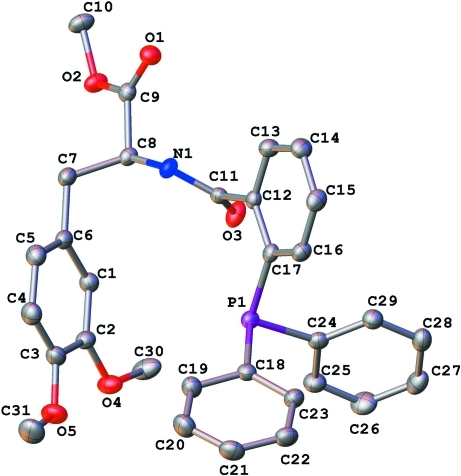
The molecular structure of the title compound. Displacement ellipsoids are drawn at the 40% probability level. Hydrogen atoms have been omitted for clarity.

**Fig. 2.**
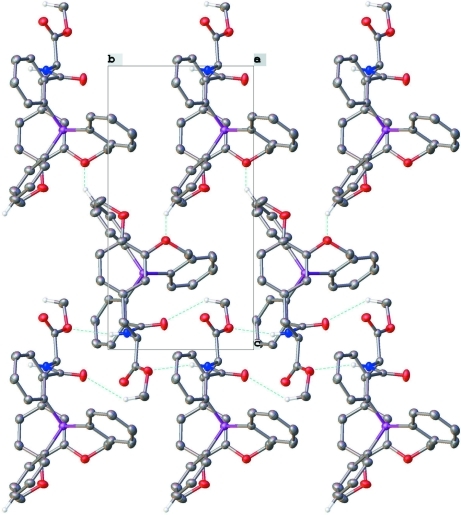
A view of packing of the the title compound along the a axis.

## supplementary crystallographic information

### Comment

The title compound is being used as a precusor to novel chiral organocatalysts (Naicker *et al.* 2010 and 2011). Analogous structures are well known precusors to several biologically active compounds (Zalán *et al.*, 2006).

There is an analogous X-ray crystal structure reported (Clegg and Elsegood, 2003), which has a *tert*-butoxy group at the ester carboxyl carbon and a (9-*H*-Fluoren-9-yl)-methoxy group attached to the amide carboxyl carbon. The title compound has a methoxy and a 2-diphenylphoshinobenzene group at the these positions respectively.

The title compound exists in a well ordered staggered conformation about the C7—C8 bond (Fig. 1). As in the analogous X-ray structure, the dimethoxybenzene ring is *gauche* to the amide group and *anti*  to the ester group. The configuration at C8 was confirmed to be *S*, on the basis of anomalous scaterring effects, Flack *x*  parameter = -0.08 (6).

The molecules in the crystal are connected by relatively weak hydrogen bond interactions (Fig. 2) in which the N1—H1···O2 and the C10—H10A···O3 interactions give chains along the *b* axis. These chains are interconnected *via* the C21—H21···O4 interaction giving a layered packing system.

### Experimental

2-(diphenylphosphanyl)benzoic acid (1.3 g, 4.2 mmol) was dissolved was dissolved in DMF (15 ml) and THF (5 ml) followed by addition of HBTU (4.6 mmol), DIPEA (8.4 mmol) and (*S*)-methyl 2-amino-3-(3,4-dimethoxyphenyl)propanoate (1.0 g, 4.2 mmol). The reaction mixture was then stirred at room temperature until no more starting material could be detected by TLC analysis. The reaction mixture was poured into 30 volumes of chilled water; the mixture was then extracted thrice with ethyl acetate (20 ml). The combined extracts were dried over anhydrous sodium sulfate and then concentrated to dryness affording the crude product. This crude product was purified by column chromatography (50:50 EtOAc/Hexane, *R*_f_ = 0.6) to afford the product 2.20 g (98%) as a white solid. *M*.p. = 420 K.

Recrystallization from ethyl acetate at room temperature afforded crystals suitable for X-ray analysis.

### Refinement

All non-hydrogen atoms were refined anisotropically. All hydrogen atoms could be found in the difference electron density maps. H1N was thus positioned and refined freely with independent isotropic temperature factors. The other hydrogen atoms were placed with idealized positions and refined as riding on their parents atoms with *U*_iso_ = 1.2 or 1.5 times *U*_eq_ (C).

### Crystal data

**Table:**

C_31_H_30_NO_5_P	*F*(000) = 556
*M**_r_* = 527.53	*D*_x_ = 1.307 Mg m^−^^3^
Monoclinic, *P*2_1_	Melting point: 420 K
Hall symbol: P 2yb	Mo *K*α radiation, λ = 0.71073 Å
*a* = 10.2218 (3) Å	Cell parameters from 6654 reflections
*b* = 8.4535 (2) Å	θ = 2.6–28.3°
*c* = 15.7633 (4) Å	µ = 0.14 mm^−^^1^
β = 100.300 (2)°	*T* = 173 K
*V* = 1340.16 (6) Å^3^	Needle, colourless
*Z* = 2	0.18 × 0.15 × 0.14 mm

### Data collection

**Table:**

Nonius KappaCCD diffractometer	5550 reflections with *I* > 2σ(*I*)
Radiation source: fine-focus sealed tube	*R*_int_ = 0.000
graphite	θ_max_ = 28.3°, θ_min_ = 2.6°
1.2° φ scans and ω scans	*h* = −13→13
6654 measured reflections	*k* = −11→11
6654 independent reflections	*l* = −21→20

### Refinement

**Table:**

Refinement on *F*^2^	Secondary atom site location: difference Fourier map
Least-squares matrix: full	Hydrogen site location: inferred from neighbouring sites
*R*[*F*^2^ > 2σ(*F*^2^)] = 0.034	H atoms treated by a mixture of independent and constrained refinement
*wR*(*F*^2^) = 0.079	*w* = 1/[σ^2^(*F*_o_^2^) + (0.0449*P*)^2^ + 0.0315*P*] where *P* = (*F*_o_^2^ + 2*F*_c_^2^)/3
*S* = 1.04	(Δ/σ)_max_ < 0.001
6654 reflections	Δρ_max_ = 0.17 e Å^−^^3^
350 parameters	Δρ_min_ = −0.25 e Å^−^^3^
1 restraint	Absolute structure: Flack (1983), 3108 Friedel pairs
Primary atom site location: structure-invariant direct methods	Flack parameter: −0.08 (6)

### Special details

**Table:**

Geometry. All e.s.d.'s (except the e.s.d. in the dihedral angle between two l.s. planes) are estimated using the full covariance matrix. The cell e.s.d.'s are taken into account individually in the estimation of e.s.d.'s in distances, angles and torsion angles; correlations between e.s.d.'s in cell parameters are only used when they are defined by crystal symmetry. An approximate (isotropic) treatment of cell e.s.d.'s is used for estimating e.s.d.'s involving l.s. planes.
Refinement. Refinement of *F*^2^ against ALL reflections. The weighted *R*-factor *wR* and goodness of fit *S* are based on *F*^2^, conventional *R*-factors *R* are based on *F*, with *F* set to zero for negative *F*^2^. The threshold expression of *F*^2^ > σ(*F*^2^) is used only for calculating *R*-factors(gt) *etc*. and is not relevant to the choice of reflections for refinement. *R*-factors based on *F*^2^ are statistically about twice as large as those based on *F*, and *R*- factors based on ALL data will be even larger.

### Fractional atomic coordinates and isotropic or equivalent isotropic displacement parameters (Å^2^)

**Table:**

	*x*	*y*	*z*	*U*_iso_*/*U*_eq_	
P1	0.92652 (4)	0.26037 (5)	0.25790 (2)	0.02523 (9)	
O1	0.48837 (10)	0.38280 (13)	−0.10867 (6)	0.0317 (2)	
O2	0.32444 (10)	0.23354 (13)	−0.07247 (7)	0.0307 (2)	
O3	0.74192 (10)	0.13486 (13)	0.08701 (8)	0.0350 (3)	
O4	0.59931 (12)	0.12392 (14)	0.36882 (7)	0.0368 (3)	
O5	0.60041 (13)	0.38582 (16)	0.45360 (7)	0.0418 (3)	
N1	0.63147 (12)	0.36602 (16)	0.05671 (8)	0.0245 (3)	
H1N	0.6418 (16)	0.461 (2)	0.0513 (10)	0.028 (5)*	
C1	0.50466 (14)	0.2509 (2)	0.23296 (9)	0.0284 (3)	
H1	0.5024	0.1538	0.2024	0.034*	
C2	0.55163 (14)	0.2534 (2)	0.32072 (9)	0.0285 (3)	
C3	0.55354 (16)	0.3961 (2)	0.36673 (10)	0.0315 (4)	
C4	0.51056 (17)	0.5329 (2)	0.32311 (11)	0.0349 (4)	
H4	0.5119	0.6301	0.3535	0.042*	
C5	0.46479 (16)	0.52901 (19)	0.23368 (11)	0.0334 (4)	
H5	0.4364	0.6242	0.2040	0.040*	
C6	0.46036 (14)	0.38986 (19)	0.18847 (9)	0.0275 (3)	
C7	0.40679 (15)	0.3851 (2)	0.09251 (9)	0.0295 (3)	
H7A	0.3922	0.4949	0.0710	0.035*	
H7B	0.3195	0.3310	0.0828	0.035*	
C8	0.49880 (13)	0.30031 (16)	0.03990 (9)	0.0239 (3)	
H8	0.5042	0.1861	0.0567	0.029*	
C9	0.43998 (14)	0.31191 (17)	−0.05562 (9)	0.0243 (3)	
C10	0.24347 (15)	0.2574 (2)	−0.15711 (10)	0.0361 (4)	
H10A	0.2296	0.3709	−0.1677	0.054*	
H10B	0.1573	0.2051	−0.1595	0.054*	
H10C	0.2889	0.2123	−0.2012	0.054*	
C11	0.74258 (13)	0.27876 (18)	0.07843 (8)	0.0227 (3)	
C12	0.86721 (14)	0.37492 (18)	0.08839 (9)	0.0226 (3)	
C13	0.88991 (15)	0.4619 (2)	0.01690 (9)	0.0288 (3)	
H13	0.8264	0.4589	−0.0351	0.035*	
C14	1.00365 (17)	0.5522 (2)	0.02102 (10)	0.0332 (4)	
H14	1.0202	0.6074	−0.0285	0.040*	
C15	1.09289 (15)	0.56158 (19)	0.09765 (11)	0.0318 (4)	
H15	1.1694	0.6269	0.1016	0.038*	
C16	1.07142 (14)	0.47595 (19)	0.16908 (10)	0.0281 (3)	
H16	1.1339	0.4838	0.2213	0.034*	
C17	0.96012 (13)	0.37840 (18)	0.16613 (9)	0.0235 (3)	
C18	1.01378 (14)	0.37421 (19)	0.35058 (9)	0.0266 (3)	
C19	0.93471 (16)	0.4673 (2)	0.39468 (10)	0.0344 (4)	
H19	0.8411	0.4685	0.3760	0.041*	
C20	0.99036 (18)	0.5573 (2)	0.46472 (11)	0.0420 (4)	
H20	0.9349	0.6192	0.4940	0.050*	
C21	1.12645 (18)	0.5579 (2)	0.49259 (11)	0.0390 (4)	
H21	1.1648	0.6214	0.5403	0.047*	
C22	1.20615 (16)	0.4658 (2)	0.45073 (10)	0.0361 (4)	
H22	1.2997	0.4655	0.4700	0.043*	
C23	1.15084 (15)	0.3736 (2)	0.38064 (10)	0.0321 (3)	
H23	1.2067	0.3093	0.3528	0.039*	
C24	1.04146 (15)	0.09383 (18)	0.25728 (9)	0.0263 (3)	
C25	1.01978 (17)	−0.0371 (2)	0.30662 (10)	0.0346 (4)	
H25	0.9461	−0.0375	0.3358	0.042*	
C26	1.10386 (19)	−0.1664 (2)	0.31385 (12)	0.0435 (4)	
H26	1.0882	−0.2541	0.3483	0.052*	
C27	1.21046 (17)	−0.1684 (2)	0.27115 (11)	0.0395 (4)	
H27	1.2688	−0.2568	0.2768	0.047*	
C28	1.23209 (16)	−0.0417 (2)	0.22019 (11)	0.0351 (4)	
H28	1.3042	−0.0439	0.1897	0.042*	
C29	1.14867 (15)	0.0889 (2)	0.21343 (10)	0.0313 (4)	
H29	1.1646	0.1760	0.1786	0.038*	
C30	0.6152 (2)	−0.0166 (2)	0.32221 (12)	0.0446 (4)	
H30A	0.5276	−0.0568	0.2954	0.067*	
H30B	0.6613	−0.0964	0.3617	0.067*	
H30C	0.6676	0.0066	0.2774	0.067*	
C31	0.6213 (2)	0.5309 (2)	0.50066 (12)	0.0485 (5)	
H31A	0.6819	0.5985	0.4751	0.073*	
H31B	0.6603	0.5085	0.5609	0.073*	
H31C	0.5360	0.5854	0.4982	0.073*	

### Atomic displacement parameters (Å^2^)

**Table:**

	*U*^11^	*U*^22^	*U*^33^	*U*^12^	*U*^13^	*U*^23^
P1	0.02244 (17)	0.02806 (19)	0.02497 (18)	−0.00083 (17)	0.00365 (14)	0.00163 (17)
O1	0.0317 (6)	0.0349 (6)	0.0290 (5)	−0.0030 (5)	0.0067 (5)	0.0031 (5)
O2	0.0272 (5)	0.0309 (6)	0.0307 (5)	−0.0069 (5)	−0.0039 (4)	0.0041 (5)
O3	0.0266 (6)	0.0204 (6)	0.0570 (8)	0.0016 (5)	0.0046 (5)	0.0029 (5)
O4	0.0513 (7)	0.0302 (6)	0.0272 (6)	0.0053 (5)	0.0022 (5)	0.0015 (5)
O5	0.0531 (7)	0.0453 (7)	0.0255 (6)	0.0012 (6)	0.0026 (5)	−0.0058 (5)
N1	0.0206 (6)	0.0188 (7)	0.0326 (7)	−0.0002 (5)	0.0005 (5)	0.0012 (5)
C1	0.0275 (7)	0.0292 (8)	0.0285 (7)	−0.0016 (7)	0.0048 (6)	−0.0028 (7)
C2	0.0280 (7)	0.0297 (8)	0.0275 (7)	0.0004 (7)	0.0040 (6)	0.0050 (7)
C3	0.0304 (8)	0.0375 (9)	0.0266 (8)	−0.0006 (7)	0.0052 (7)	−0.0031 (7)
C4	0.0378 (9)	0.0319 (9)	0.0357 (9)	0.0024 (7)	0.0085 (7)	−0.0056 (7)
C5	0.0354 (9)	0.0298 (9)	0.0362 (9)	0.0037 (7)	0.0092 (7)	0.0025 (7)
C6	0.0213 (7)	0.0329 (8)	0.0285 (8)	0.0010 (6)	0.0051 (6)	0.0030 (7)
C7	0.0246 (7)	0.0343 (8)	0.0293 (8)	0.0039 (7)	0.0040 (6)	0.0050 (7)
C8	0.0204 (7)	0.0225 (8)	0.0279 (8)	−0.0023 (6)	0.0016 (6)	0.0029 (6)
C9	0.0234 (7)	0.0201 (7)	0.0290 (8)	0.0004 (6)	0.0038 (6)	−0.0008 (6)
C10	0.0340 (8)	0.0364 (8)	0.0324 (8)	−0.0055 (8)	−0.0090 (6)	0.0034 (8)
C11	0.0243 (7)	0.0253 (8)	0.0186 (6)	−0.0001 (6)	0.0038 (5)	−0.0019 (6)
C12	0.0203 (7)	0.0222 (7)	0.0251 (7)	0.0025 (6)	0.0037 (6)	−0.0029 (6)
C13	0.0289 (8)	0.0311 (8)	0.0258 (8)	−0.0007 (7)	0.0031 (6)	0.0029 (7)
C14	0.0317 (8)	0.0358 (9)	0.0334 (9)	−0.0033 (7)	0.0089 (7)	0.0071 (7)
C15	0.0233 (7)	0.0306 (9)	0.0413 (9)	−0.0051 (7)	0.0048 (7)	0.0054 (7)
C16	0.0233 (7)	0.0296 (8)	0.0297 (8)	−0.0016 (7)	0.0002 (6)	0.0010 (7)
C17	0.0207 (7)	0.0221 (7)	0.0275 (7)	0.0033 (6)	0.0036 (6)	0.0000 (6)
C18	0.0280 (8)	0.0285 (8)	0.0232 (7)	0.0004 (7)	0.0045 (6)	0.0028 (6)
C19	0.0305 (8)	0.0414 (9)	0.0318 (9)	0.0010 (8)	0.0068 (7)	−0.0020 (8)
C20	0.0456 (10)	0.0476 (11)	0.0342 (9)	0.0021 (9)	0.0111 (8)	−0.0087 (8)
C21	0.0453 (10)	0.0445 (10)	0.0257 (8)	−0.0064 (9)	0.0022 (7)	−0.0053 (8)
C22	0.0325 (8)	0.0438 (10)	0.0296 (8)	−0.0029 (8)	−0.0014 (7)	0.0015 (8)
C23	0.0283 (8)	0.0382 (9)	0.0291 (8)	0.0029 (7)	0.0031 (6)	0.0028 (7)
C24	0.0273 (8)	0.0278 (8)	0.0220 (7)	−0.0023 (6)	−0.0003 (6)	−0.0017 (6)
C25	0.0392 (9)	0.0328 (9)	0.0320 (8)	−0.0003 (8)	0.0069 (7)	0.0050 (7)
C26	0.0565 (11)	0.0281 (9)	0.0454 (10)	0.0043 (8)	0.0079 (9)	0.0089 (8)
C27	0.0393 (9)	0.0325 (9)	0.0437 (10)	0.0092 (8)	−0.0012 (8)	−0.0057 (8)
C28	0.0294 (8)	0.0384 (9)	0.0372 (9)	0.0030 (8)	0.0049 (7)	−0.0069 (8)
C29	0.0317 (8)	0.0323 (9)	0.0302 (8)	0.0002 (7)	0.0063 (7)	0.0030 (7)
C30	0.0605 (12)	0.0277 (9)	0.0394 (10)	0.0049 (9)	−0.0076 (9)	0.0004 (8)
C31	0.0547 (12)	0.0533 (12)	0.0361 (10)	−0.0007 (10)	0.0049 (9)	−0.0177 (9)

### Geometric parameters (Å, °)

**Table:**

P1—C24	1.8348 (16)	C13—H13	0.9500
P1—C17	1.8395 (15)	C14—C15	1.380 (2)
P1—C18	1.8413 (15)	C14—H14	0.9500
O1—C9	1.2046 (17)	C15—C16	1.389 (2)
O2—C9	1.3387 (18)	C15—H15	0.9500
O2—C10	1.4529 (17)	C16—C17	1.399 (2)
O3—C11	1.2242 (18)	C16—H16	0.9500
O4—C2	1.371 (2)	C18—C23	1.396 (2)
O4—C30	1.421 (2)	C18—C19	1.399 (2)
O5—C3	1.3703 (19)	C19—C20	1.377 (2)
O5—C31	1.430 (2)	C19—H19	0.9500
N1—C11	1.3462 (19)	C20—C21	1.382 (2)
N1—C8	1.4454 (18)	C20—H20	0.9500
N1—H1N	0.82 (2)	C21—C22	1.377 (2)
C1—C2	1.381 (2)	C21—H21	0.9500
C1—C6	1.401 (2)	C22—C23	1.388 (2)
C1—H1	0.9500	C22—H22	0.9500
C2—C3	1.406 (2)	C23—H23	0.9500
C3—C4	1.377 (2)	C24—C25	1.393 (2)
C4—C5	1.404 (2)	C24—C29	1.397 (2)
C4—H4	0.9500	C25—C26	1.383 (2)
C5—C6	1.372 (2)	C25—H25	0.9500
C5—H5	0.9500	C26—C27	1.380 (3)
C6—C7	1.514 (2)	C26—H26	0.9500
C7—C8	1.538 (2)	C27—C28	1.380 (3)
C7—H7A	0.9900	C27—H27	0.9500
C7—H7B	0.9900	C28—C29	1.388 (2)
C8—C9	1.521 (2)	C28—H28	0.9500
C8—H8	1.0000	C29—H29	0.9500
C10—H10A	0.9800	C30—H30A	0.9800
C10—H10B	0.9800	C30—H30B	0.9800
C10—H10C	0.9800	C30—H30C	0.9800
C11—C12	1.496 (2)	C31—H31A	0.9800
C12—C13	1.399 (2)	C31—H31B	0.9800
C12—C17	1.4101 (19)	C31—H31C	0.9800
C13—C14	1.383 (2)		
			
C24—P1—C17	101.67 (7)	C13—C14—H14	120.3
C24—P1—C18	100.69 (7)	C14—C15—C16	120.29 (15)
C17—P1—C18	102.03 (7)	C14—C15—H15	119.9
C9—O2—C10	116.81 (12)	C16—C15—H15	119.9
C2—O4—C30	116.35 (12)	C15—C16—C17	121.72 (14)
C3—O5—C31	117.21 (14)	C15—C16—H16	119.1
C11—N1—C8	123.87 (13)	C17—C16—H16	119.1
C11—N1—H1N	116.5 (12)	C16—C17—C12	117.29 (13)
C8—N1—H1N	119.6 (12)	C16—C17—P1	123.97 (11)
C2—C1—C6	120.78 (16)	C12—C17—P1	118.72 (11)
C2—C1—H1	119.6	C23—C18—C19	117.87 (14)
C6—C1—H1	119.6	C23—C18—P1	125.55 (12)
O4—C2—C1	124.56 (15)	C19—C18—P1	116.58 (11)
O4—C2—C3	115.41 (12)	C20—C19—C18	121.10 (15)
C1—C2—C3	120.03 (15)	C20—C19—H19	119.4
O5—C3—C4	125.14 (15)	C18—C19—H19	119.4
O5—C3—C2	115.56 (15)	C19—C20—C21	120.34 (16)
C4—C3—C2	119.31 (14)	C19—C20—H20	119.8
C3—C4—C5	120.05 (15)	C21—C20—H20	119.8
C3—C4—H4	120.0	C22—C21—C20	119.57 (16)
C5—C4—H4	120.0	C22—C21—H21	120.2
C6—C5—C4	121.06 (15)	C20—C21—H21	120.2
C6—C5—H5	119.5	C21—C22—C23	120.49 (16)
C4—C5—H5	119.5	C21—C22—H22	119.8
C5—C6—C1	118.76 (14)	C23—C22—H22	119.8
C5—C6—C7	120.94 (14)	C22—C23—C18	120.61 (15)
C1—C6—C7	120.29 (14)	C22—C23—H23	119.7
C6—C7—C8	113.84 (12)	C18—C23—H23	119.7
C6—C7—H7A	108.8	C25—C24—C29	118.02 (14)
C8—C7—H7A	108.8	C25—C24—P1	116.15 (11)
C6—C7—H7B	108.8	C29—C24—P1	125.82 (12)
C8—C7—H7B	108.8	C26—C25—C24	121.04 (15)
H7A—C7—H7B	107.7	C26—C25—H25	119.5
N1—C8—C9	110.39 (11)	C24—C25—H25	119.5
N1—C8—C7	111.48 (12)	C27—C26—C25	120.21 (16)
C9—C8—C7	109.39 (11)	C27—C26—H26	119.9
N1—C8—H8	108.5	C25—C26—H26	119.9
C9—C8—H8	108.5	C26—C27—C28	119.80 (16)
C7—C8—H8	108.5	C26—C27—H27	120.1
O1—C9—O2	124.44 (13)	C28—C27—H27	120.1
O1—C9—C8	125.49 (13)	C27—C28—C29	120.14 (15)
O2—C9—C8	110.06 (12)	C27—C28—H28	119.9
O2—C10—H10A	109.5	C29—C28—H28	119.9
O2—C10—H10B	109.5	C28—C29—C24	120.76 (15)
H10A—C10—H10B	109.5	C28—C29—H29	119.6
O2—C10—H10C	109.5	C24—C29—H29	119.6
H10A—C10—H10C	109.5	O4—C30—H30A	109.5
H10B—C10—H10C	109.5	O4—C30—H30B	109.5
O3—C11—N1	123.47 (13)	H30A—C30—H30B	109.5
O3—C11—C12	123.36 (13)	O4—C30—H30C	109.5
N1—C11—C12	113.14 (13)	H30A—C30—H30C	109.5
C13—C12—C17	120.39 (13)	H30B—C30—H30C	109.5
C13—C12—C11	117.51 (12)	O5—C31—H31A	109.5
C17—C12—C11	122.10 (12)	O5—C31—H31B	109.5
C14—C13—C12	120.76 (14)	H31A—C31—H31B	109.5
C14—C13—H13	119.6	O5—C31—H31C	109.5
C12—C13—H13	119.6	H31A—C31—H31C	109.5
C15—C14—C13	119.45 (14)	H31B—C31—H31C	109.5
C15—C14—H14	120.3		
			
C30—O4—C2—C1	7.8 (2)	C12—C13—C14—C15	−2.7 (2)
C30—O4—C2—C3	−171.77 (14)	C13—C14—C15—C16	2.6 (3)
C6—C1—C2—O4	−178.59 (14)	C14—C15—C16—C17	0.0 (2)
C6—C1—C2—C3	1.0 (2)	C15—C16—C17—C12	−2.4 (2)
C31—O5—C3—C4	−8.0 (2)	C15—C16—C17—P1	179.37 (12)
C31—O5—C3—C2	171.56 (14)	C13—C12—C17—C16	2.3 (2)
O4—C2—C3—O5	−1.2 (2)	C11—C12—C17—C16	−178.21 (13)
C1—C2—C3—O5	179.23 (13)	C13—C12—C17—P1	−179.38 (11)
O4—C2—C3—C4	178.39 (15)	C11—C12—C17—P1	0.11 (18)
C1—C2—C3—C4	−1.2 (2)	C24—P1—C17—C16	−77.33 (14)
O5—C3—C4—C5	179.85 (15)	C18—P1—C17—C16	26.42 (14)
C2—C3—C4—C5	0.3 (2)	C24—P1—C17—C12	104.47 (12)
C3—C4—C5—C6	0.8 (2)	C18—P1—C17—C12	−151.78 (11)
C4—C5—C6—C1	−1.1 (2)	C24—P1—C18—C23	26.80 (15)
C4—C5—C6—C7	178.01 (14)	C17—P1—C18—C23	−77.71 (15)
C2—C1—C6—C5	0.2 (2)	C24—P1—C18—C19	−152.39 (12)
C2—C1—C6—C7	−178.91 (13)	C17—P1—C18—C19	103.10 (12)
C5—C6—C7—C8	130.11 (15)	C23—C18—C19—C20	0.9 (2)
C1—C6—C7—C8	−50.83 (19)	P1—C18—C19—C20	−179.80 (14)
C11—N1—C8—C9	−109.43 (14)	C18—C19—C20—C21	0.4 (3)
C11—N1—C8—C7	128.76 (13)	C19—C20—C21—C22	−1.1 (3)
C6—C7—C8—N1	−53.92 (17)	C20—C21—C22—C23	0.4 (3)
C6—C7—C8—C9	−176.30 (13)	C21—C22—C23—C18	0.9 (3)
C10—O2—C9—O1	−10.2 (2)	C19—C18—C23—C22	−1.6 (2)
C10—O2—C9—C8	168.85 (12)	P1—C18—C23—C22	179.23 (13)
N1—C8—C9—O1	−8.7 (2)	C17—P1—C24—C25	−165.63 (12)
C7—C8—C9—O1	114.33 (16)	C18—P1—C24—C25	89.57 (13)
N1—C8—C9—O2	172.30 (12)	C17—P1—C24—C29	15.66 (15)
C7—C8—C9—O2	−64.66 (15)	C18—P1—C24—C29	−89.14 (14)
C8—N1—C11—O3	−0.8 (2)	C29—C24—C25—C26	1.7 (2)
C8—N1—C11—C12	177.40 (12)	P1—C24—C25—C26	−177.15 (14)
O3—C11—C12—C13	119.27 (17)	C24—C25—C26—C27	−0.8 (3)
N1—C11—C12—C13	−58.97 (17)	C25—C26—C27—C28	−0.8 (3)
O3—C11—C12—C17	−60.23 (19)	C26—C27—C28—C29	1.4 (3)
N1—C11—C12—C17	121.52 (14)	C27—C28—C29—C24	−0.5 (2)
C17—C12—C13—C14	0.2 (2)	C25—C24—C29—C28	−1.0 (2)
C11—C12—C13—C14	−179.31 (14)	P1—C24—C29—C28	177.64 (12)

### Hydrogen-bond geometry (Å, °)

**Table:**

*D*—H···*A*	*D*—H	H···*A*	*D*···*A*	*D*—H···*A*
N1—H1N···O2^i^	0.816 (17)	2.345 (17)	3.1428 (17)	166 (15)
C10—H10A···O3^i^	0.98	2.56	3.371 (2)	140
C21—H21···O4^ii^	0.95	2.58	3.279 (2)	131

Symmetry codes: (i) −*x*+1, *y*+1/2, −*z*; (ii) −*x*+2, *y*+1/2, −*z*+1.
